# Hybrid Polymer-Network Hydrogels with Tunable Mechanical Response

**DOI:** 10.3390/polym8030082

**Published:** 2016-03-15

**Authors:** Sebastian Czarnecki, Torsten Rossow, Sebastian Seiffert

**Affiliations:** 1 Institute of Chemistry and Biochemistry, Freie Universität Berlin, Takustr. 3, D-14195 Berlin, Germany; sebastian.seiffert@helmholtz-berlin.de (S.C.); torsten.rossow@onlinehome.de (T.R.); 2 Soft Matter and Functional Materials, Helmholtz-Zentrum Berlin, Hahn-Meitner-Platz 1, D-14109 Berlin, Germany

**Keywords:** hybrid polymer hydrogel, mechanical fuse links, model-network structure, poly(ethylene glycol)

## Abstract

Hybrid polymer-network gels built by both physical and covalent polymer crosslinking combine the advantages of both these crosslinking types: they exhibit high mechanical strength along with excellent fracture toughness and extensibility. If these materials are extensively deformed, their physical crosslinks can break such that strain energy is dissipated and irreversible fracturing is restricted to high strain only. This mechanism of energy dissipation is determined by the kinetics and thermodynamics of the physical crosslinking contribution. In this paper, we present a poly(ethylene glycol) (PEG) based material toolkit to control these contributions in a rational and custom fashion. We form well-defined covalent polymer-network gels with regularly distributed additional supramolecular mechanical fuse links, whose strength of connectivity can be tuned without affecting the primary polymer-network composition. This is possible because the supramolecular fuse links are based on terpyridine–metal complexation, such that the mere choice of the fuse-linking metal ion adjusts their kinetics and thermodynamics of complexation–decomplexation, which directly affects the mechanical properties of the hybrid gels. We use oscillatory shear rheology to demonstrate this rational control and enhancement of the mechanical properties of the hybrid gels. In addition, static light scattering reveals their highly regular and well-defined polymer-network structures. As a result of both, the present approach provides an easy and reliable concept for preparing hybrid polymer-network gels with rationally designed properties.

## 1. Introduction

Polymer hydrogels consist of water-swollen macromolecular networks with a soft consistency that resembles that of native tissue. They can be formed by either permanent-covalent or by transient-physical polymer crosslinking, which strongly influences their mechanical properties. Physically crosslinked hydrogels are often stimuli-responsive [1,2] and self-healing [3], but they do not exhibit high mechanical strength and often exhibit plastic flow. By contrast, covalently crosslinked hydrogels exhibit a permanently fixed shape at rest, but they usually have low fracture toughness and extensibility. To create hydrogels that combine all the preceding advantages, both physical and covalent polymer crosslinking must be combined [4,5,6]. This combination results in doubly crosslinked hybrid gels; if these are strongly deformed, their physical crosslinks can break such that strain energy is dissipated and irreversible fracturing is restricted to occurring at very high strain; this concept has also been applied to elastomers [7]. The mechanism of energy dissipation is determined by the kinetics and thermodynamics of the physical crosslinking contribution; hence, the pursuit of rational design of such doubly crosslinked hybrid hydrogels requires a comprehensive understanding of this interplay. This endeavor, however, is complicated by the fact that the physical-chemical characteristics of the physical crosslinks in a doubly crosslinked hybrid network cannot be supposed to be the same as in a pure physical network [6]. Hence, to derive such knowledge, model systems are needed that fulfill two requirements: First, the covalent network must exhibit a well-defined topology without defects or inhomogeneities [8]. Second, the binding strength and binding-unbinding dynamics of the physical crosslinking must be tunable independent of the covalent crosslinking.

A useful example of a dually crosslinked hybrid gel-network system has been introduced by Narita and collaborators [6,9]. These researchers reported on poly(vinylalcohol) (PVA) hydrogels with both chemical and physical crosslinks [10,11], which they studied via macro- and microrheology as well as by dynamic light scattering in comparison to plain chemical and plain physical PVA gels. In the dually-crosslinked hybrid gels, an associative Rouse mode characterized by equivalent power-law scaling of the frequency-dependent storage and loss shear moduli, *G*′ = *G*′′ ~ ω^0.5^, was observed in rheology, and a three-dimensional finite-strain constitutive model was developed to quantify this rate-dependent mechanical behavior and the kinetics of breaking and reattachment of the temporary physical crosslinks [12]. However, the authors also observed deviation of their experimentally determined scaling exponents from classical theoretical predictions, which they attributed to network imperfection caused by loops, dangling chains, and larger spatial inhomogeneities due to PVA hydrogen-bonding; this is in accord with our own previous related results [13,14]. In addition to this shortcoming, another limitation of this system is the restriction of its physical crosslinking to just one single type, which is borate esterification, thereby preventing the strength and dynamics of the physical crosslinking fraction to be varied and tuned. In another recent example, Zhou and coworkers developed a dually crosslinked hybrid hydrogel wherein the physical crosslinking is based on acrylic-iron(III) coordination [5]. In this example, the supramolecular bonds again strongly enhance the gel mechanical properties by opening a path for energy dissipation. However, in this approach, the covalent network was prepared by radical copolymerization, and despite its simplicity, this strategy has the drawback that the resulting network is often very inhomogeneous [8]. This inhomogeneity affects the properties of both the covalent and the supramolecular parts of the network and therefore prevents this system from being used to derive general structure–property relations of dually crosslinked hybrid networks. To overcome these existing limitations, Sakai and coworkers introduced a dually-crosslinked polymer gel of very homogeneous network architecture based on tetra-arm star-shaped poly(ethylene glycol) (PEG) as well as linear PEG and poly(dimethylsiloxane) (PDMS) building blocks linked together by orthogonal cross-coupling, thereby forming a network wherein hydrophilic and hydrophobic components are distributed regularly and uniformly [15]. In an aqueous environment, aggregated hydrophobic pDMS segments serve as non-covalent “mechanical fuse links” that are capable of avoiding sudden macroscopic fracture. In this approach, the non-covalent hydrophobic association strength is tuned by the molar ratio of the hydrophilic PEG and the hydrophobic PDMS segments; this principle, however, comes along with the drawback of change of the overall network composition upon variation of this ratio, which impairs independent variation of the transient crosslinking strength to rationally tune the network mechanics. Thus, a more sophisticated model system should consist of both a well-defined homogenous covalent [16,17,18] and a well-defined homogeneous physical network component [19], and it should allow the kinetics and thermodynamics of the latter to be tuned in an independent and versatile fashion without affecting the network composition and architecture.

**Figure 1:**
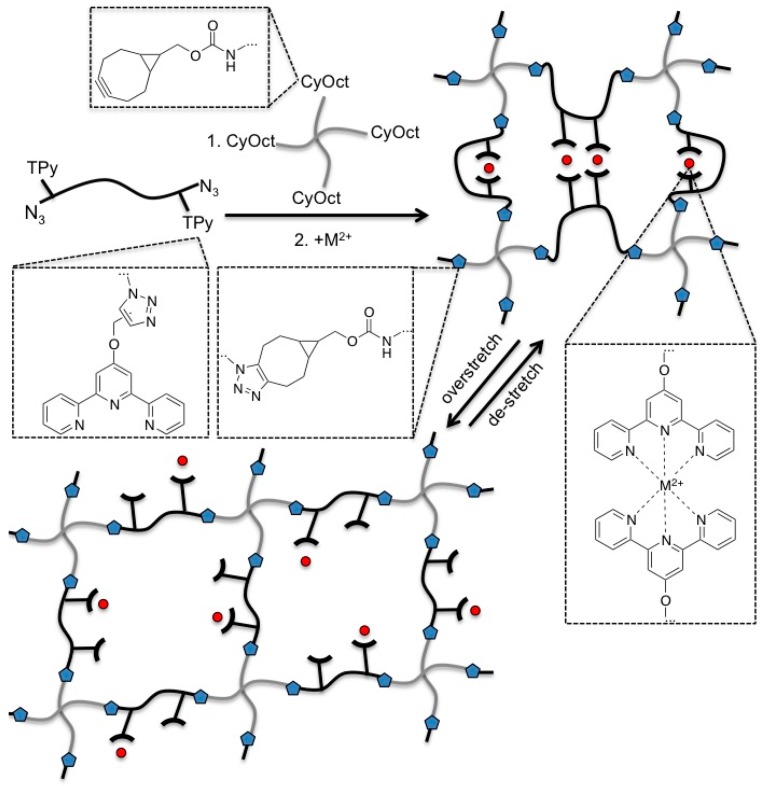
Formation of hybrid polymer-network gels that contain both covalent and physical crosslinks. For this purpose, linear poly(ethylene glycol) (PEG) is functionalized with both azide (N_3_) and terpyridine (TPy) moieties at each end, and tetra-arm PEG is functionalized with cyclooctynes (CyOct) capped to each arm. After mixing aqueous solutions of both these PEG compounds, gelation occurs by strain-promoted azide–alkyne cycloaddition forming triazole linking units sketched as blue pentagons in the schematic. Addition of metal(II) ions, sketched as red circles in the schematic, leads to formation of intramolecular or intermolecular mechanical fuse links due to complexation to the additional terpyridine moieties on the polymer. Upon stretching, these hybrid gel-networks first respond in a linear rubbery-elastic fashion, before overstretching results in breakage of the transient metal–terpyridine bis-complexes, thereby dissipating strain energy, while leaving the covalent polymer crosslinking intact. Upon de-stretching, the metal–terpyridine bis-complexes are reformed. The illustrated hybrid polymer-network is drawn in an idealized fashion without loops or dangling chains; the data presented in this work indeed support this ideal picture and suggest the minor extent of such imperfection.

In this paper, we build upon Sakai’s recent work [15] and present a material toolkit to form well-defined covalent polymer-network gels with regularly distributed supramolecular mechanical fuse links, whose strength of connectivity can be tuned without affecting the polymer-network composition. In addition, this hybrid network has the potential to exhibit close-to regular and well-defined nanometer-scale network topologies for both the covalent network and the supramolecular component. To realize this goal, linear poly(ethylene glycol) (PEG) is functionalized with both azide (N_3_) and terpyridine (TPy) moieties at each end, and additional tetra-arm star-shaped PEG is functionalized with cyclooctynes (CyOct) capped to each arm. Upon mixing aqueous solutions of both these PEG compounds, gelation occurs by strain-promoted azide–alkyne cycloaddition [20,21], thereby forming a homogeneous covalent tetra-PEG-type network [16,17,18], as shown in Figure 1. Addition of metal(II) ions such as Co^2+^, Zn^2+^, or Mn^2+^ leads to additional formation of intramolecular or intermolecular mechanical fuse links due to complexation to the terpyridine moieties on the polymer [22,23,24,25,26], as also shown in Figure 1. This design principle allows the effects of the thermodynamics and kinetics of the metal-complexation-based mechanical fuse links on the dual network mechanics to be tuned [27] and investigated systematically, based upon kinetic and thermodynamic data determined by UV spectroscopy [24] and isothermal titration calorimetry (ITC) [19], as assembled in Table 1. We use oscillatory shear rheology to relate these tunable fuse-linking characteristics to the macroscopic frequency- and amplitude-dependent mechanical properties of the resulting hybrid gels. Furthermore, we use static light scattering to probe the highly regular topology of the hybrid polymer networks. These combined studies demonstrate the utility of this material platform to form hybrid polymer-network gels with tunable mechanical properties in a consistent, rational, and versatile fashion.

## 2. Materials and Methods

### 2.1. General Remarks

^1^H-NMR spectra were recorded at 300 K in either CDCl_3_ or DMSO-d_6_ on a Bruker (Billerica, MA, USA) DPX 400, a Bruker AVANCE 500 (Billerica, MA, USA), or a Bruker AVANCE 700 (Billerica, MA, USA), with TMS (δ_H_ = 0.00 ppm) serving as an internal reference in each case. Attenuated total reflection infrared (ATR-IR) spectra were recorded on a JASCO FT/IR–4600 (Easton, MD, USA). Chemicals were purchased from Sigma Aldrich (St. Louis, MO, USA) and used without further purification. Tetra-arm hydroxy-terminated PEG (*M*_w_ = 10 kg·mol^−1^, *M*_w_/*M*_n_ = 1.05) was purchased from Creative PEGWorks (Chapel Hill, NC, USA), whereas linear epoxy-terminated PEG (*M*_w_ = 2.5 kg·mol^−1^, *M*_w_/*M*_n_ = 1.12) was purchased from Sigma Aldrich (St. Louis, MO, USA). Dry dimethylformamide, dry acetonitrile, and dry pyridine were purchased from Acros Chemicals (Geel, Belgium), whereas dry dichloromethane, dry tetrahydrofuran, and dry diethyl ether were obtained from a MB SPS-800 solvent purification system (Stratham, NH, USA). Triethylamine was dried over CaH_2_ and distilled before use in reactions. All aqueous solutions were saturated unless specified differently. All reactions were conducted under anhydrous argon and monitored by thin-layer chromatography on Kieselgel 60 F254 purchased from Merck (Kenilworth, NJ, USA), if not mentioned differently. Detection of the spots was carried out by staining the TLC plates with aqueous KMnO_4_. Silica gel (Merck Kieselgel 60 0.015–0.040 mm) was used for column chromatography. For dialysis of tetra-arm PEG-bicyclo[6.1.0]non-4-yn-9-ylmethyl carbamate (**9**), benzoylated dialysis tubing (avg. flat width 32 mm) purchased from Sigma Aldrich was used, whereas metal-free dialysis tubing (nom. flat width 45 mm) purchased from Spectra/Por was used for dialysis of tetra-arm PEG-terpyridinyl-azide to ensure absence of metal ions that could complex to the terpyridine motifs during the dialysis. Tetra-arm PEG-amine (**7**) was prepared according to a procedure of Elbert and Hubbell [28]. Bicyclo[6.1.0]non-4-yn-9-ylmethyl (4-nitrophenyl) carbonate (**8**) was synthesized according to a procedure of van Delft and co-workers [29].

### 2.2. Synthesis

PEG-hydroxy-azide (**1**). Sodium azide (3.25 g, 50 mmol) was carefully added to a solution of PEG-epoxide (5 g, 2.5 mmol) in dimethylformamide (100 mL), followed by addition of ammonium chloride (5.35 g, 100 mmol). The mixture was stirred at 60 °C for 48 h. The suspension was filtered to remove remaining sodium azide and ammonium chloride, and the filtrate was concentrated *in vacuo*. The residue was dissolved in a mixture of dichloromethane (100 mL) and brine (50 mL) and extracted with dichloromethane (2 × 200 mL). The organic layer was dried over NaSO_4_, concentrated *in vacuo*, and the product was precipitated by pouring the concentrate into cold (0 °C) diethyl ether. The precipitate was collected by filtration and washed with cold diethyl ether to obtain the product as a white solid (4.37 g, 87.6%). ATR-IR (cm^−1^): 2881 (m), 2739 (w), 2360 (w), 2341 (w), 2099 (m), 1465 (m), 1412 (w), 1359 (w), 1341 (m), 1278 (m), 1240 (m), 1145 (m), 1103 (s), 1059 (s), 957 (s), 946 (s), 840 (s). ^1^H-NMR (DMSO-d_6_, 700 MHz): δ = 5.24 (s, 2H), 3.77 (s, 2H), 3.61 (dd, *J* = 5.8, 4.0 Hz, 2H), 3.31–3.22 (m, 212H), 3.20 (d, *J* = 6.6 Hz, 2H), 3.19 (d, *J* = 6.6 Hz, 2H) ppm.

Propargyl-terpyridine (**2**). Dried potassium carbonate (6.65 g, 48.1 mmol) was suspended in acetonitrile (120 mL). 4′-hydroxy-2,2′-6′,6′′-terpyridine (2 g, 8.0 mmol) was added to this suspension, followed by addition of 3-bromoprop-1-yne (1 mL, 9.0 mmol). The brick-red colored suspension was stirred at 60 °C for 15 h. The product was precipitated by pouring the suspension into cold deionized water. The precipitate was collected by filtration, washed thoroughly with cold water, and recrystallized from hexane/ethyl acetate (1:2) to obtain pale-brown fine needles (1.81 g, 82.5%). ^1^H-NMR (DMSO-d_6_, 400 MHz): δ = 8.77–8.69 (m, 2H), 8.63 (d, *J* = 7.9 Hz, 2H), 8.04 (s, 2H), 8.01 (td, 2H), 7.51 (ddd, 2H), 5.11 (d, *J* = 2.4 Hz, 2H), 3.72 (t, *J* = 2.4 Hz, 1H) ppm.

PEG-hydroxy-terpyridine (**3**). 4-(2-propyn-1-oxy)-2,2-6,6-terpyridine (**2**) (1.89 g, 6.6 mmol) was added to a melt of the PEG-hydroxy-azide (**1**) (4.37 g, 2.2 mmol). The melt was stirred at 90 °C for 12 h, allowed to cool down to room temperature and was then dissolved in dichloromethane (20 mL). The solution was precipitated in cold (0 °C) diethyl ether, and the precipitated solid was collected by filtration and washed with cold diethyl ether to obtain the product as a white solid (4.56 g, 79.5%). ATR-IR (cm^−1^): 3416 (w), 2881 (m), 2740 (w), 2361 (w), 2341 (w), 1965 (w), 1599 (w), 1581 (m), 1563 (m), 1466 (m), 1406 (w), 1358 (m), 1342 (m), 1279 (m), 1240 (m), 1198 (w), 1145 (m), 1059 (s), 1011 (m), 960 (m), 841 (m), 795 (m), 745 (w), 735 (w), 693 (w), 658 (w). ^1^H-NMR (DMSO-d_6_, 500 MHz): δ = 8.75–8.70 (m, 4H), 8.65–8.59 (m, 4H), 8.18 (s, 1H), 7.97 (d, *J* = 1.8 Hz, 4H), 7.85 (s, 1H), 7.63–7.46 (m, 4H), 5.58 (s, 1H), 5.42 (s, 2H), 5.33 (d, *J* = 5.6 Hz, 1H), 5.28 (d, *J* = 5.5 Hz, 1H), 4.69–4.22 (m, 2H), 3.52–3.48 (m, 212H) ppm.

PEG-terpyridinyl-*p*-nitrophenylcarbonate (**4**). PEG-hydroxy-terpyridine (**3**) (4.56 g, 2.3 mmol) was dissolved in dichloromethane (40 mL). Pyridine was added to this solution (1.1 mL, 13.7 mmol), followed by further addition of *p*-nitrophenyl chloroformate (1.44 g, 6.9 mmol). The suspension was stirred at room temperature for 12 h, the precipitate was filtered off, and brine (100 mL) was added to the filtrate. The mixture was extracted with dichloromethane (2 × 200 mL), the organic layer was dried over MgSO_4_ and then concentrated *in vacuo*. The product was precipitated by pouring the concentrate into cold (0 °C) diethyl ether to obtain the product as a white solid (3.05 g, 45%). ^1^H- NMR (DMSO-d_6_, 700 MHz): δ = 8.75–8.68 (m, 4H), 8.65–8.60 (m, 4H), 8.34 (s, 1H), 8.28 (d, *J* = 9.1 Hz, 2H), 8.09 (s, 2H), 8.08 (s, 2H), 8.00 (td, 4H), 7.96 (s, 1H), 7.52–7.48 (m, 4H), 7.42 (d, *J* = 9.1 Hz, 2H), 7.39 (d, *J* = 9.2 Hz, 2H), 5.49 (s, 2H), 4.94–4.76 (m, 2H), 3.83–3.74 (m, 4H), 3.52–3.48 (m, 212H) ppm.

PEG-terpyridinyl-azide (**5**). Triethylamine (430 µL, 3 mmol) was added to a solution of the PEG-terpyridinyl-*p*-nitrophenyl carbonate (**4**) (3.05 g, 1.0 mmol) in dimethylformamide (10 mL, HPLC grade), followed by further addition of *O*-(2-Aminoethyl)-*O*′-(2-azidoethyl)nonaethylene glycol (1.19 g, 2.2 mmol). The brown mixture was stirred overnight at room temperature, purified by dialysis, and lyophilized to isolate a brownish sticky solid (2.70 g, 91%). IR-ATR (cm^−1^): 2864 (m), 2360 (m), 2341 (m), 2102 (m), 1599 (w), 1581 (w), 1562 (m), 1467 (m), 1405 (w), 1348 (m), 1294 (m), 1248 (m), 1189 (w), 1093 (s), 1037 (s), 946 (m), 846 (m), 798 (m), 747 (m), 693 (m), 669 (m), 639 (m). ^1^H-NMR (DMSO-d_6_, 700 MHz): δ = 8.73 (m, 4H), 8.63 (m, 4H), 8.22 (s, 2H), 8.10 (s, 2H), 8.09 (s, 1H), 8.01 (m, 4H), 7.88 (s, 1H), 7.55-7.49 (m, 4H), 5.61 (s, 2H), 5.44 (s, 2H), 5.22–5.16 (m, 1H), 5.14–5.09 (m, 1H), 4.78–4.56 (m, 2H), 3.52–3.48 (m, 248H) ppm.

Tetra-arm PEG-bicyclo[6.1.0]non-4-yn-9-ylmethyl carbamate (**8**). Triethylamine (170 µL, 1.2 mmol) was added to a solution of tetra-arm PEG-amine 7 (1 g, 0.1 mmol) in dimethylformamide (10 mL, HPLC grade), followed by addition of bicyclo[6.1.0]non-4-yn-9-ylmethyl (4-nitrophenyl) carbonate 8 (0.14 g, 0.44 mmol). The yellow solution was stirred for 12 h at room temperature, purified by dialysis in water, and lyophilized to obtain a white solid (1.05 g, 98%). ^1^H-NMR (DMSO-d_6_, 700 MHz): δ = 3.52–3.48 (m, 902H), 3.54–3.43 (m, 24H), 0.86 (m, 2H), 0.70–0.60 (m, 6H) ppm.

### 2.3. Oscillatory Shear Rheology

Rheological studies were conducted on a stress-controlled Anton Paar Physica MCR 301 rheometer with a parallel plate–plate geometry (gap size 1 mm, plate diameter 25 mm) and a strain-controlled Rheometrics Fluids Spectrometer II with an equal geometry. For sample preparation, an aqueous semidilute solution of the tetra-arm PEG-bicyclo[6.1.0]non-4-yn-9-ylmethyl carbamate (**8**) was distributed evenly in the middle of the lower plate at 25 °C, and the upper geometry was lowered to evenly distribute the polymer solution on the upper geometry, too. The upper geometry was then elevated again, and an aqueous semidilute solution of linear PEG-terpyridinyl-azide (**5**) was added to the polymer solution on the lower plate, instantly followed by addition of an aqueous solution of the metal-crosslinker, and then the upper geometry was lowered again. The polymer and metal-ion concentrations in the three preceding sets of solutions were chosen to yield a final polymer content of 100 g·L^−1^ in each sample, realized by an equimolar concentration of both added polymer solutions, and a stoichiometric amount of metal ions relative to the concentration of terpyridine groups, respectively. For the first 60 min, each sample was monitored at a constant shearing amplitude and frequency (γ = 0.1; ω = 0.01 Hz) to ensure sample equilibration. Then, a frequency sweep was recorded at constant strain amplitude (γ = 0.1; ω = 0.01–16 Hz) at 25 °C. After decreasing the temperature to 10 °C and allowing for equilibration for another 20 min, a second frequency sweep was recorded. Thereafter, the temperature was increased to 25 °C again, allowing the sample to equilibrate for another 20 min, and an amplitude sweep was recorded at a constant frequency (γ = 0.1–1000; ω = 0.01 Hz). In case of the Zn^2+^–TPy-system in water, a second set of measurements was conducted starting with a 60-min equilibration period at 25 °C followed by an amplitude sweep at constant frequency (γ = 0.1–140; ω = 0.01 Hz). Then, the system was allowed to re-equilibrate at 25 °C for 30 min, and a frequency sweep at constant strain amplitude (γ = 0.1; ω = 0.01–16 Hz) was recorded.

### 2.4. Static Light Scattering

Static inhomogeneities of the nanometer-scale polymer segmental density in the different hybrid-gels that arise as a consequence of potentially inhomogeneous chain crosslinking were probed by static light scattering. For this purpose, 2 mL of each hybrid hydrogel at a total polymer concentration of 100 g·L^−1^ and, as a reference, an uncrosslinked PEG-OH solution in water at the same concentration were prepared in cylindrical NMR tubes (Wilmad Labglass) with an inner diameter of 1 cm. All solutions were filtered through a 25-µm PDMS filter to minimize dust within them. Scattering intensities of these four different samples were estimated at detection angles between 20° and 140° in 10°-intervals using a ALV 125 goniometer and a HeNe laser (λ = 632 nm, *T* = 25 °C). To calculate the excess light scattering intensities of the different hybrid polymer networks, *R*_E_(*q*), the scattering intensities of the uncrosslinked PEG-OH sample, *R*_Sol_(*q*), were subtracted from that of the hybrid crosslinked polymer gels, *R*_Gel_(*q*). Analyzing the excess scattering intensities with the Debye-Bueche method [30,31,32] yields the static correlation length and the root-mean-square fluctuation of the refractive index, which can be transferred into mean-square concentration fluctuations [33] if the refractive-index increment is known. These values can then serve as a measure of the relative polymer-network inhomogeneity.

## 3. Results and Discussion

### 3.1. Polymer Synthesis and Hybrid Network Formation

**Scheme 1:**
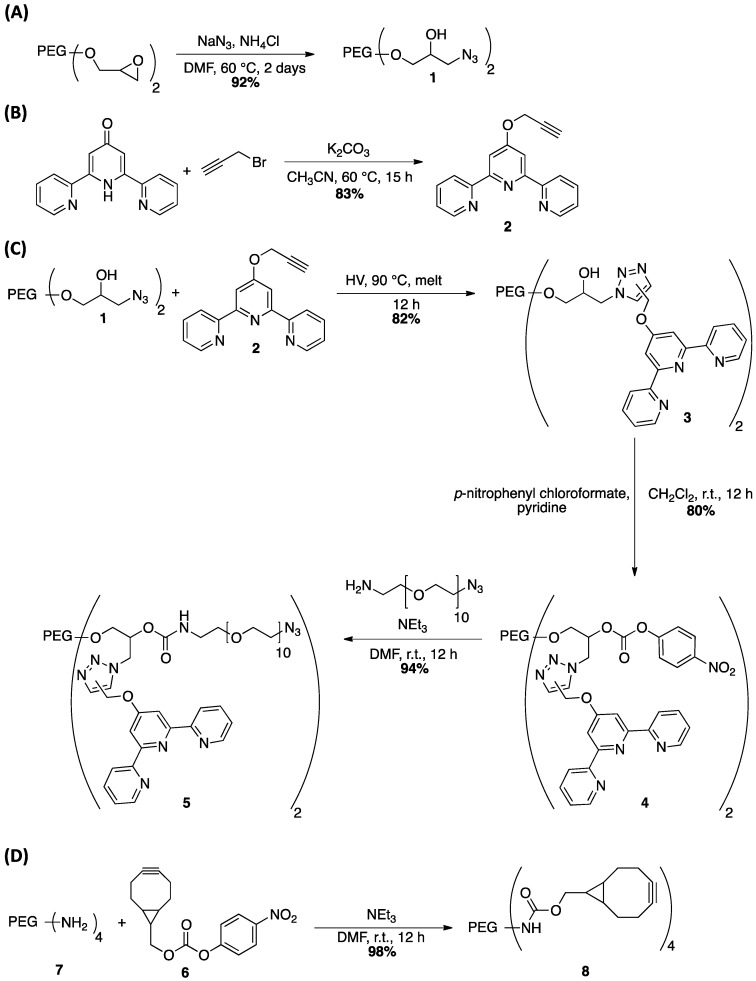
Synthesis of (**A**) linear PEG-azide (**1**), (**B**) propargyl-terpyridine (**2**); (**C**) linear PEG-terpyridinyl-azide (**5**); and (**D**) tetra-arm PEG-bicyclo[6.1.0]non-4-yn-9-ylmethyl carbamate (**8**).

**Figure 2:**
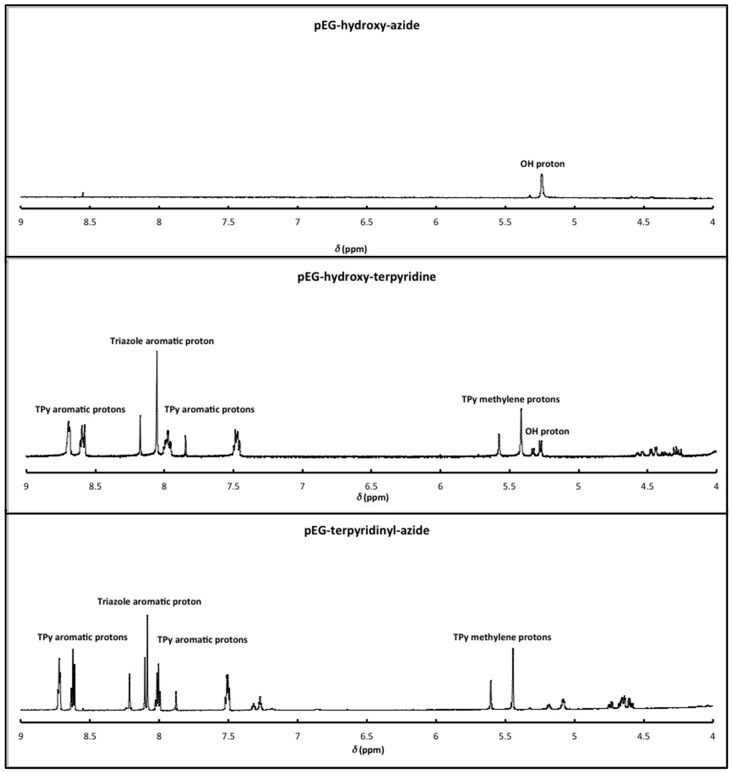
^1^H-NMR spectra of PEG-hydroxy-azide (**top**), PEG-hydroxy-terpyridine (**center**) and PEG-terpyridinyl-azide (**bottom**) in a chemical-shift range of 4–9 ppm, where the signals of the compounds differ most significantly.

Our approach to form hybrid polymer gels with both supramolecular and covalent polymer crosslinking is based on two commercially available narrowly distributed poly(ethylene glycol) (PEG) building blocks, tetra-arm PEG-OH and linear PEG-epoxide, both derivatized to display the complementary pattern of chain-end functionality illustrated in Figure 1. For this purpose, the tetra-arm PEG-OH is functionalized to carry cyclooctyne moieties end-capped to each of its arms, whereas the linear PEG-epoxide is functionalized to carry both azide and terpyridine moieties at each end, as shown in Figure 1. To be able to attach two different functional groups to each end of the linear PEG-epoxide, this polymer is first converted with sodium azide, resulting in epoxide-ring opening and formation of azide-terminated linear PEG with two additional OH-groups (**1**), as sketched in Scheme 1A and proven by ^1^H-NMR spectroscopy (Figure 2). Then, terpyridine motifs are attached to this polymer in an azide-alkyne Huisgen cycloaddition, which is a simple and quantitative click reaction [34,35]. For this purpose, propargyl-terpyridine (**2**) is prepared by reaction of 2,6-bis(2-pyridyl)-4-pyridone with propargyl bromide [36], as illustrated in Scheme 1B. The azide-alkyne Huisgen cycloaddition is then conducted at 90 °C in a melt of 1 under high vacuum, thereby avoiding PEG decomposition. The resulting triazole-product is obtained as a mixture of both regio-isomers in a ratio of 3:1 (1,4-adduct:1,5-adduct) determined by ^1^H-NMR spectroscopy (Figure 2). We convert the remaining OH-groups with *p*-nitrophenyl chloroformate to obtain PEG-terpyridinyl-*p*-nitrophenolcarbonate (**4**), which is subsequently reacted with *O*-(2-aminoethyl)-*O*′-(2-azidoethyl)nonaethylene glycol to form the carbamate 5, as illustrated in Scheme 1C and proven by ^1^H-NMR spectroscopy (Figure 2). The short PEG-unit between the terpyridine side groups and the adjacent azide end groups on each chain end acts as a spacer to prevent shielding of the azides by the bulky terpyridines. For preparation of the second building block, we convert tetra-arm PEG-OH into tetra-arm PEG-amine (**7**) by mesylation and subsequent substitution with ammonia [28]. Then, we react 7 with bicyclo[6.1.0]non-4-yn-9-ylmethyl (4-nitrophenyl) carbonate (**6**), which we previously prepared according to a procedure of van Delft and co-workers [29], resulting in PEG-bicyclo[6.1.0]non-4-yn-9-ylmethyl carbamate (**8**), as shown in Scheme 1D. Both PEG building blocks can be synthesized with a multigram yield. This set of two PEG-based building blocks allows hybrid polymer-network gels with both covalent crosslinks and additional supramolecular metal-complexation-type mechanical fuse links to be formed. In these hybrid gels, the strength of the supramolecular fuse-linking can be varied by the choice of the complexing metal(II) ion. We use the d-row of the periodic table as a toolbox and pick Zn(NO_3_)_2_, Co(NO_3_)_2_, and Mn(NO_3_)_2_ as the complexing metal salts, because these salts are known to form complexes of markedly varying strength with terpyridine [22,23,24,25,26]. A quantity that represents the supramolecular binding affinity of these complexes is their association equilibrium constant, *K*, which relates the equilibrium concentration of associated complexes to that of the non-associated binding motifs. As terpyridine binds to metal(II) ions in a 2:1 ratio (M + 2L → ML_2_), K = [ML^2^]/([M][L]^2^). A second quantity represents the supramolecular binding and unbinding *kinetics* of these complexes in the form of their dissociation rate constants, *k*_diss._. These two quantities, as determined in previous works [19,24], are detailed in Table 1. The binding affinity increases from Mn^2+^ to Co^2+^ and Zn^2+^, whereas the kinetic stability of the bisterpyridine complexes decreases from Co^2+^ to Zn^2+^ and Mn^2+^. These trends reflect the Irving–Williams and spectrochemical series [37], which can be reasoned within the concept of crystal field stabilization energy.

### 3.2. Gel-Network Mechanics

**Figure 3:**
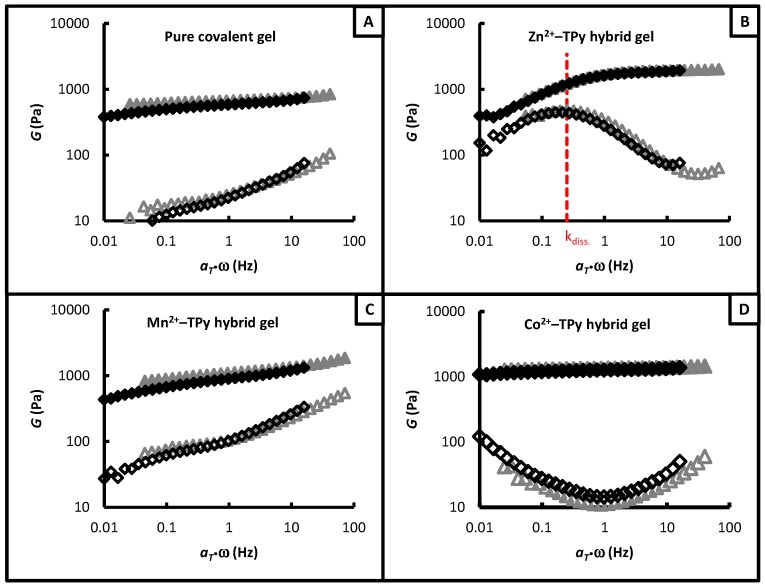
Frequency-dependent shear moduli of the (**A**) purely covalent gel and the (**B**) Zn^2+^-, (**C**) Mn^2+^-; and (**D**) Co^2+^-terpyridine-enforced hybrid gels, each probed at a total polymer concentration of 100 g L^−1^ in water. All plots show the elastic (full symbols, *G*′) and viscous (open symbols, *G*′′) parts of the complex shear modulus, *G*, as a function of the test frequency, ω. Grey triangles denote data obtained at 10 °C, while black diamonds denote data obtained at 25 °C. All data are superimposed by shifting the 10 °C data along the frequency axes to obtain master curves according to the principle of time-temperature-superposition, referenced to 25 °C. The vertical dashed red line in (**B**) denotes ω = k*_diss._* of the Zn^2+^–terpyridine complexes.

To demonstrate the utility of the hybrid gels obtained by the present approach, we use oscillatory shear rheology to determine their elastic and viscous shear moduli, *G*′ and *G*′′. For this purpose, solutions of the polymers and the interlinking metal ions are prepared in water and then mixed in a rheometer, resulting in instantaneous formation of the transient physical fuse links and the covalent network in each case. To ensure that every terpyridine moiety can be complexed in the gels, stoichiometric amounts of metal nitrate relative to the terpyridine moieties are added. The polymer concentration in each sample is 100 g·L^−1^; this corresponds to a polymer volume fraction of ϕ = 0.075 and is close to the polymer overlap concentration of 133 g·L^−1^ [19]. In the rheometer, each sample is left to react while monitored with a constant weak shearing amplitude of 0.1 at a frequency of 0.01 Hz for 60 min; during this time, *G*′ and *G*′′ slowly reach a plateau, which we refer to good sample homogenization before and during the gelation and to the presence of an equilibrium state afterwards. Then, frequency sweeps are recorded at 10 and 25 °C, and additional amplitude sweeps are recorded at 25 °C at the end of each measurement protocol, thereby disrupting the gel. The data of *G*′ and *G*′′ can be shifted along the frequency axis to obtain master curves referenced to *T* = 25 °C, as depicted in Figure 3. For comparison, Figure 3 also contains the rheological data of the corresponding purely covalent gel without addition of any metal ions (Panel A). The pure chemical gel displays a purely elastic response with an elastic modulus *G*′ that is independent of the frequency and larger than the viscous modulus *G*′′(ω). The Zn^2+^-based hybrid gel, by contrast, exhibits a larger viscous modulus showing a peak at ω = 0.25 Hz; this frequency matches the dissociation rate constant k*_diss._* of the Zn^2+^–terpyridine complex in Table 1, as highlighted by the vertical dashed red line in Figure 3B. We identify two different regimes characterized by two different rubbery elastic plateaus, one in the frequency regime below and one in the frequency range above ω = k*_diss._*, denoted as *G*_p_* and *G*_p_, respectively, in Table 2. *G*_p_* is identical to the plateau value of the pure covalent polymer gel, as also listed in Table 2. This finding suggests that in the low-frequency limit, the supramolecular mechanical fuse-links are inactive such that the hybrid gel has the same crosslinking density as the pure covalent gel, composed by its own covalent crosslinking fraction only.

By contrast, the plateau values in the high-frequency limit suggest that in this frequency range, the mechanical fuse links are active and therefore provide an additional mechanically active component in the gel. Base on this premise, the difference *G*_p_ − *G*_p_* is assumed to reflect the elastic contribution by the fuse-link component. We use the estimated values of *G*_p_ and *G*_p_ − *G*_p_* to determine the efficiency of formation of covalent crosslinks and of additional physical fuse links according to the phantom-network model [38]. This model links the plateau modulus to the concentration of elastically effective tetrafunctional crosslinks in the gel, *v* = *G*_P_/(ϕ*RT*)_,_ with *T* the temperature, *R* the gas constant, and ϕ the polymer volume fraction. For the purely covalent gel, we calculate the concentration of elastically effective crosslinks to be *v* = 4.8 mmol·L^−1^. In an ideal homogeneous network build-up of star-shaped precursors, each star provides an elastically effective tetrafunctional crosslinking node, and thus *v*_theo_ = 5 mmol·L^−1^ denotes the ideal value of *v* in the case of fully efficient and homogeneous crosslinking of the present type of precursor polymers. The experimental estimate of *v* in the covalent gel is in excellent agreement with this ideal case, indicating such efficient and homogenous crosslinking. For the additional fuse-link contribution in the Zn^2+^-hybrid gel, we calculate *v* = 5.6 mmol·L^−1^, which is in good agreement to the concentration of Zn^2+^–terpyridine complexes in this gel, *c* = 5.5 mmol·L^−1^. In a simplifying view, this finding indicates efficient formation of the mechanical fuse links in the Zn^2+^-hybrid gel, wherein each of the fuse links acts like one additional elastically effective crosslinking junction. This is a remarkable finding, because the fuse-linking does not form additional elastically effective network chains, but just further ties the strands of existing meshes together in a form of multiple crosslinking [39,40,41,42,43,44]. This process, however, reduces the network-strand flexibility and conformational freedom, and the result of this reduction manifests itself in form of an additional entropy-elastic contribution to the network modulus [44].

In contrast to the two-plateau rheological characteristics of the Zn^2+^-hybrid gel, the Mn^2+^-hybrid gel does not exhibit any rubbery elastic plateau, but a gradual increase of both *G*′ and *G*′′ in the frequency range probed, as seen in Figure 3C. We presume that the kinetically labile Mn^2+^-terpyridine complexes result in hybrid gels with a fully established *G*_P_ only at frequencies higher than ω > 100 Hz. Thus, the gradually increasing *G*′(ω) and *G*′′(ω) of the Mn^2+^-hybrid gel are interpreted to correspond to a frequency regime of transition between the low-frequency and the high-frequency plateaus. By contrast, the Co^2+^-hybrid gel exhibits a frequency-independent rubbery elastic plateau in the full frequency regime tested, as seen in Figure 3D. This is due to the high kinetic stability of the Co^2+^-terpyridine complexes, which do not break on the timescale probed in rheology but are expected to do so only at frequencies ω < 0.01 Hz [24]. Hence, these gels display the covalent-plus-fuse-link plateau *G*_P_ in the whole frequency range probed. Based on this picture, this modulus is expected to be the same as the one found in the high-frequency domain of the Zn^2+^-hybrid gel. In contrast to this expectation, however, we find *G*_P_ of the Co^2+^-hybrid gel to be slightly smaller, as quantified in Table 2. A potential reason for this discrepancy can be less efficient enforcement of the gel mechanics by the Co^2+^-complexes. This may be due to different extents of secondary interactions between the complexes in the Zn^2+^- and the Co^2+^-hybrid gels, for example, in the form of different extent of clustering, which has been concluded to provide favorable mechanical enforcement in one of our earlier studies [19]. To check for the first possibility, we probe the network nanostructures by dynamic light scattering, as detailed in Section 3.3.

To further probe the utility of the metal-complexation-based fuse links for improvement of the mechanical fracture toughness of the hydrogels, amplitude sweeps were recorded in a strain range of 0.1%–1000% at 25 °C to determine the yield strength, γ_yield_, and the rupture strength, γ_rupture_, of the materials, as summarized in Table 3. The yield strength is defined as the strain at which the elastic modulus starts to decrease, whereas the rupture strength is defined as the strain at which the material collapses, denoted by crossover of *G*′ and *G*′′ in the amplitude sweeps.

The Mn^2+^-hybrid gel exhibits improved stability compared to the pure covalent reference gel, which is assessed by prolongation of its linear viscoelastic regime by a factor of 1.4. By contrast, both hybrid gels containing metal ions with higher strength of complexation, Zn^2+^ and Co^2+^, are more rigid and yield even earlier than the pure covalent reference gel, even though in these gels the rupture points are shifted to higher strain amplitudes with increasing strength of metal complexation, from Zn^2+^ to Co^2+^. This finding suggests that the Mn^2+^-hybrid gel exhibits a lower number of mechanically active fuse links in comparison to the Zn^2+^- and Co^2+^-hybrid gel, resulting in a gel that has a similar flexibility to the purely covalent gel but that is capable of dissipating strain energy. By contrast, fuse links based on strongly binding Co^2+^-links result in a decreased stability of the system due to less network flexibility, but exhibit improved rupture strength due to the higher number of mechanically active fuse links.

**Figure 4:**
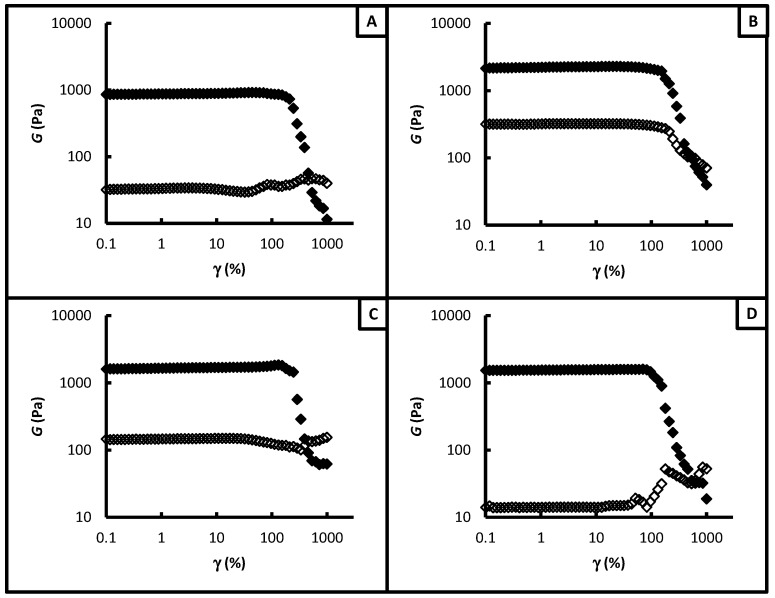
Amplitude-dependent shear moduli of the (**A**) purely covalent gel and the (**B**) Zn^2+^-, (**C**) Mn^2+^-; and (**D**) Co^2+^-terpyridine-enforced hybrid gels, each probed at a total polymer concentration of 100 g L^−1^ in water. All plots show the elastic (full symbols, *G*′) and viscous (open symbols, *G*′′) parts of the complex shear modulus, *G*, as a function of the shear-deformation amplitude, γ, at 25 °C.

To verify our initial hypothesis that the metal–terpyridine complexes are capable of recovering upon destretching of the material, a second amplitude sweep was conducted in a second set of rheology experiments. In this case, the applied strain deformation during the first amplitude sweep was limited to not exceed the determined yield stress of the hybrid gel, and the strain was then removed and the hybrid gels allowed to rest for 30 min. Afterwards, a second frequency sweep was conducted. When the results of these tests are compared to the data depicted in Figure 4, full identity is revealed.

### 3.3. Network Nanostructures

To demonstrate the high regularity of the covalent-and-supramolecular polymer networks in the three hybrid gels, the samples were probed by static light scattering in conjunction with data evaluation with the Debye–Bueche method [30,31,32,33]. In this approach, it is assumed that the scattering intensity of a gel sums up thermal concentration fluctuations (ergodic contributions) and static spatial inhomogeneities resulting from the covalent and supramolecular crosslinking (nonergodic contribution).

**Figure 5:**
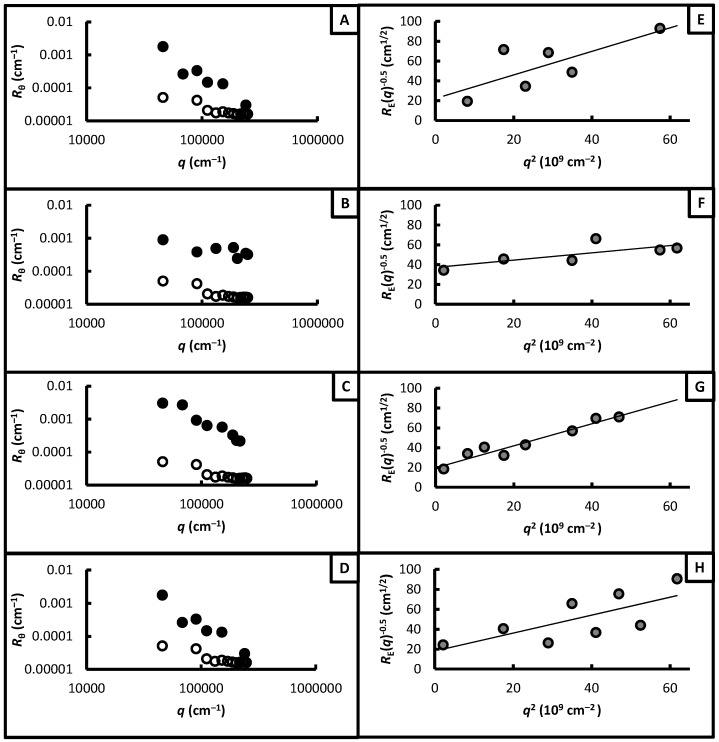
Static light scattering data used to evaluate the spatial polymer network inhomogeneity of the (**A,E**) purely covalent gel and of the (**B**,**F**) Zn^2+^-, (**C**,**G**) Mn^2+^-, and (**D**,**H**) Co^2+^-terpyridine-enforced hybrid gels, each probed at a total polymer concentration of 100 g·L^−1^ in water. (**A**–**D**) Rayleigh ratios, *R*_θ_, of the gels (full symbols) and of an uncrosslinked reference sample (open symbols); (**E**–**H**) Debye-Bueche plots of the excess scattering intensities, *R*_E_(*q*), calculated as the difference between the angle-resolved Rayleigh ratios of the crosslinked and uncrosslinked samples.

To determine the value of the latter, it is further assumed that the thermal fluctuations are equivalent to those in an uncrosslinked solution of the same polymer at the same concentration [39,40,41,42,43,44]. Thus, the excess scattering intensity of a polymer gel can be determined from the difference of the angle-resolved scattering between a crosslinked and uncrosslinked sample [30,33] which are both shown in Figure 5A–D. Subsequent linearization of the data by the Debye-Bueche method, as shown in Figure 5E–H, yields two characteristic network parameters: the static correlation length and the root-mean-square refractive index fluctuation. If the refractive index increment is known, the latter fluctuations can be converted into concentration fluctuations [33]. We follow this approach and determine a relative concentration fluctuation of 1.5% in the pure covalent gel, of 3.1% in the Zn^2+^-hybrid gel, of 1.9% in the Co^2+^-hybrid gel, and of 1.6% in the Mn^2+^-hybrid gel. All these gels are therefore highly homogeneous and exhibit only weak irregularities on the nanometer scale in a general view. In a more specific view, the Zn^2+^-hybrid gel is found to be slightly more inhomogeneous than the Co^2+^-hybrid gel. In one of our earlier studies [19], the opposite order has been found for related pure supramolecular gels crosslinked by these ions in dimethylformamide medium, and agreement to this earlier study, the more inhomogeneous gel (here: the Zn^2+^-hybrid hydrogel; earlier study: the pure supramolecular Co^2+^ organogel) displays a stronger elastic modulus, as quantified in Table 2. Hence, just as in our earlier study [19], we conclude that slight inhomogeneity seems to enforce a supramolecular or partly supramolecular polymer-network gel.

## 4. Conclusions

The polymer-network construction toolkit presented in this paper allows model-type hybrid hydrogels to be formed with exquisite control of both their covalent and physical network components. The gel-network mechanics can be tuned through the choice of fuse-linking metal ion, directly related to its kinetics and thermodynamics of complexation–decomplexation. In contrast to other dually crosslinked gels, the present covalent–supramolecular design principle does not affect the general composition of the polymer network, and the low polydispersity of the precursor molecular weight and architecture allows these hybrid gels to be obtained with determined and highly homogeneous nanometer-scale network topology. In these model-network gels, the supramolecular fuse links contribute to improving the mechanical properties in comparison to the corresponding purely covalent polymer gel. This enforcement is based on the capability of the metal-terpyridine complexes to dissipate strain energy and on the number of mechanically active fuse links. As a result, the PEG-based material toolkit provides an easy and reliable concept to prepare dually-crosslinked gels with close-to regular nanostructures and improved mechanical properties. Interestingly, rheological studies on these materials support an earlier finding of gel-network mechanics to be enforced by slight nanostructural inhomogeneity in fully or in partly supramolecular gels, which we aim to explore further in future work.

## Figures and Tables

**Table 1:** Equilibrium constants, K(25 °C), and dissociation rate constants, k_diss._, for the complexation and decomplexation of metal ions to methoxy-PEG-terpyridine in water, estimated by ITC [19] and UV spectroscopy [24]. Terpyridine complexes to metal(II) ions in a 2:1 ratio (M + 2L → ML_2_); as a result, K = [ML^2^]/([M][L]^2^).

Metal ion	K (L^2^·mol^−2^)	K_diss._ (s^−1^)
Zn^2+^	2.2 × 10^12^	0.25 (at 10 °C)
Co^2+^	7.2 × 10^10^	1.1 × 10^−3^ (at 25 °C)
Mn^2+^	1.3 × 10^7^	1.4 (at 10 °C)

**Table 2:** Rubbery elastic plateaus of the purely covalent gel and of the Zn^2+^-, Co^2+^-, and Mn^2+^-terpyridine-enforced hybrid gels. *G*_p_ denotes the plateau modulus in the frequency regime above ω = k*_diss._*, whereas and *G*_p_* denotes the plateau modulus in the frequency regime below.

Sample	*G*_P_ (Pa)	*G*_P_* (Pa)
covalent crosslinked gel	840	n/a
Zn^2+^-hybrid gel	1,840	850
Co^2+^-hybrid gel	1,470	not accessible
Mn^2+^-hybrid gel	not accessible	not accessible

**Table 3:** Yield strength, γ_yield_, and rupture strength, γ_rupture_ of the purely covalent gel and the Zn^2+^-, Co^2+^-, and Mn^2+^-terpyridine-enforced hybrid gels.

Sample	γ_yield_ (%)	γ_rupture_ (%)
covalent crosslinked gel	180	460
Zn^2+^-hybrid gel	150	535
Co^2+^-hybrid gel	100	625
Mn^2+^-hybrid gel	250	460

## Abbreviations

The following abbreviations are used in this manuscript:

PEG: poly(ethylene glycol)
PVA: poly(vinylalcohol)
PDMS: poly(dimethylsiloxane)

## References

[1] Wojtecki R.J., Meador M.A., Rowan S.J. (2011). Using the dynamic bond to access macroscopically responsive structurally dynamic polymers. Nat. Mater..

[2] Seiffert S., Sprakel J. (2012). Physical chemistry of supramolecular polymer networks. Chem. Soc. Rev..

[3] Cordier P., Tournilhac F., Soulie-Ziakovic C., Leibler L. (2008). Self-healing and thermoreversible rubber from supramolecular assembly. Nature.

[4] Sun J.-Y., Zhao X., Illeperuma W.R.K., Chaudhuri O., Oh K.H., Mooney D.J., Vlassak J.J., Suo Z. (2012). Highly stretchable and tough hydrogels. Nature.

[5] Lin P., Ma S., Wang X., Zhou F. (2015). Molecularly engineered dual-crosslinked hydrogel with ultrahigh mechanical strength, toughness, and good self-recovery. Adv. Mater..

[6] Narita T., Mayumi K., Ducouret G., Hébraud P. (2013). Viscoelastic properties of poly(vinyl alcohol) hydrogels having permanent and transient cross-links studied by microrheology, classical rheometry, and dynamic light scattering. Macromolecules.

[7] Tang Z., Huang J., Guo B., Zhang L., Liu F. (2016). Bioinspired engineering of sacrificial metal-ligand bonds into elastomers with supramechanical performance and adaptive recovery. Macromolecules.

[8] Di Lorenzo F., Seiffert S. (2015). Nanostructural heterogeneity in polymer networks and gels. Polym. Chem..

[9] Mayumi K., Marcellan A., Ducouret G., Creton C., Narita T. (2013). Stress-strain relationship of highly stretchable dual cross-link gels: Separability of strain and time effect. ACS Macro Lett..

[10] Shibayama M., Takeuchi T., Nomura S. (1994). Swelling/shrinking and dynamic light scattering studies on chemically cross-linked poly(vinyl alcohol) gels in the presence of borate ions. Macromolecules.

[11] Shibayama M., Uesaka M., Inamoto S., Mihara H., Nomura S. (1996). Analogy between swelling of gels and intrinsic viscosity of polymer solutions for ion-complexed poly(vinyl alcohol) in aqueous medium. Macromolecules.

[12] Long R., Mayumi K., Creton C., Narita T., Hui C.Y. (2014). Time dependent behavior of a dual cross-link self-healing gel: Theory and experiments. Macromolecules.

[13] Rossow T., Hackelbusch S., van Assenbergh P., Seiffert S. (2013). A modular construction kit for supramolecular polymer gels. Polym. Chem..

[14] Hackelbusch S., Rossow T., van Assenbergh P., Seiffert S. (2013). Chain dynamics in supramolecular polymer networks. Macromolecules.

[15] Kondo S., Hiroi T., Han Y.S., Kim T.H., Shibayama M., Chung U.I., Sakai T. (2015). Reliable hydrogel with mechanical “fuse link” in an aqueous environment. Adv. Mater..

[16] Sakai T., Matsunaga T., Yamamoto Y., Ito C., Yoshida R., Suzuki S., Sasaki N., Shibayama M., Chung U.I. (2008). Design and fabrication of a high-strength hydrogel with ideally homogeneous network structure from tetrahedron-like macromonomers. Macromolecules.

[17] Matsunaga T., Sakai T., Akagi Y., Chung U.I., Shibayama M. (2009). Structure characterization of tetra-PEG gel by small-angle neutron scattering. Macromolecules.

[18] Matsunaga T., Sakai T., Akagi Y., Chung U.I., Shibayama M. (2009). Sans and sls studies on tetra-arm PEG gels in as-prepared and swollen states. Macromolecules.

[19] Rossow T., Seiffert S. (2014). Supramolecular polymer gels with potential model-network structure. Polym. Chem..

[20] Baskin J.M., Prescher J.A., Laughlin S.T., Agard N.J., Chang P.V., Miller I.A., Lo A., Codelli J.A., Bertozzi C.R. (2007). Copper-free click chemistry for dynamic *in vivo* imaging. Proc. Natl. Acad. Sci. USA.

[21] Sletten E.M., Bertozzi C.R. (2009). Bioorthogonal chemistry: Fishing for selectivity in a sea of functionality. Angew. Chem. Int. Ed..

[22] Schubert U.S., Eschbaumer C. (2002). Macromolecules containing bipyridine and terpyridine metal complexes: Towards metallosupramolecular polymers. Angew. Chem. Int. Ed..

[23] Hogg R., Wilkins R.G. (1962). 57. Exchange studies of certain chelate compounds of the transitional metals. Part VIII. 2,2′,2′′-terpyridine complexes. J. Chem. Soc..

[24] Holyer R.H., Hubbard C.D., Kettle S.F.A., Wilkins R.G. (1966). The kinetics of replacement reactions of complexes of the transition metals with 2,2′,2′′-terpyridine. Inorg. Chem..

[25] Kim K.-Y., Nancollas G.H. (1977). Calorimetric studies of complex formation of transition metal ions with 2,2′,2′′-terpyridine. J. Phys. Chem..

[26] Constable E.C., Emeléus H.J. (1986). The coordination chemistry of 2,2′:6′,2′′-terpyridine and higher oligopyridines. Advances in Inorganic Chemistry.

[27] Henderson K.J., Zhou T.C., Otim K.J., Shull K.R. (2010). Ionically cross-linked triblock copolymer hydrogels with high strength. Macromolecules.

[28] Elbert D.L., Hubbell J.A. (2001). Conjugate addition reactions combined with free-radical cross-linking for the design of materials for tissue engineering. Biomacromolecules.

[29] Dommerholt J., Schmidt S., Temming R., Hendriks L.J.A., Rutjes F.P.J.T., van Hest J.C.M., Lefeber D.J., Friedl P., van Delft F.L. (2010). Readily accessible bicyclononynes for bioorthogonal labeling and three-dimensional imaging of living cells. Angew. Chem. Int. Ed..

[30] Debye P., Bueche A.M. (1949). Scattering by an inhomogeneous solid. J. Appl. Phys..

[31] Debye P.P. (1946). A photoelectric instrument for light scattering measurements and a differential refractometer. J. Appl. Phys..

[32] Pekeris C.L. (1947). Note on the scattering of radiation in an inhomogeneous medium. Phys. Rev..

[33] Nie J., Du B., Oppermann W. (2004). Influence of formation conditions on spatial inhomogeneities in poly(*n*-isopropylacrylamide) hydrogels. Macromolecules.

[34] Huisgen R., Padwa A. (1984). 1,3-Dipolar cycloaddition—Introduction, survey, mechanism. 1,3-Dipolar cycloaddition Chemistry.

[35] Kolb H.C., Finn M.G., Sharpless K.B. (2001). Click chemistry: Diverse chemical function from a few good reactions. Angew. Chem. Int. Ed..

[36] Armspach D., Constable E.C., Housecroft C.E., Neuburger M., Zehnder M. (1998). Carbaborane-functionalised 2,2′:6′,2″-terpyridine ligands for metallosupramolecular chemistry: Syntheses, complex formation, and the crystal and molecular structures of 4′-(ortho-carboranyl)-2,2′:6′,2″-terpyridine and 4′-(ortho-carboranylpropoxy)-2,2′:6′,2″-terpyridine1. J. Organomet. Chem..

[37] Irving H., Williams R.J.P. (1948). Order of stability of metal complexes. Nature.

[38] James H.M., Guth E. (1943). Theory of the elastic properties of rubber. J. Chem. Phys..

[39] Kizilay M.Y., Okay O. (2003). Effect of hydrolysis on spatial inhomogeneity in poly(acrylamide) gels of various crosslink densities. Polymer.

[40] Kizilay M.Y., Okay O. (2003). Effect of initial monomer concentration on spatial inhomogeneity in poly(acrylamide) gels. Macromolecules.

[41] Orakdogen N., Kizilay M.Y., Okay O. (2005). Suppression of inhomogeneities in hydrogels formed by free-radical crosslinking copolymerization. Polymer.

[42] Orakdogen N., Okay O. (2006). Correlation between crosslinking efficiency and spatial inhomogeneity in poly(acrylamide) hydrogels. Polym. Bull..

[43] Orakdogen N., Okay O. (2007). Influence of the initiator system on the spatial inhomogeneity in acrylamide-based hydrogels. J. Appl. Polym. Sci..

[44] Kuru E.A., Orakdogen N., Okay O. (2007). Preparation of homogeneous polyacrylamide hydrogels by free-radical crosslinking copolymerization. Eur. Polym. J..

